# Supplementary material for: Are carbon emissions trading and green financial instruments synergistic? -Comprehensive quantitative research based on content analysis
Source: PLoS One. 2024 Mar 7;19(3):e0298601. doi: 10.1371/journal.pone.0298601 (PMC10919601; doi:10.1371/journal.pone.0298601)
Supplement: S1 Data — (ZIP) [file pone.0298601.s001.zip › Data/地级市Z_F工作报告.zip!/║■▒▒/╗╞╕╘╩╨/└┤╫╘╣1⁄2╓┌║┼í╛┬φ┐╦╩2╛▌═°í┐_2011.docx]

各位代表：

　　现在，我代表市人民政府向大会报告政府工作，请连同《黄冈市经济和社会发展第十二个五年规划纲要（草案）》一并审议，并请市政协各位委员和其他列席人员提出意见。

　　一、“十一五”时期政府工作回顾

　　“十一五”时期是黄冈发展极不平凡的五年。在市委的正确领导下，市政府团结带领全市人民深入贯彻落实科学发展观，抢抓机遇，克难奋进，办成了一系列大事，办好了一系列喜事，保持了经济社会发展的良好态势，胜利完成了“十一五”规划确定的各项目标任务，黄冈各方面都发生了新的历史性变化。

　　过去五年，是综合实力显著提升的五年。2010年，全市地区生产总值达到862.3亿元，是“十五”末的2.4倍，年均增长14.7%。全口径财政收入99.7亿元，是“十五”末的3.4倍，年均增长27.9%，其中地方一般预算收入39亿元，是“十五”末的3倍，年均增长24.3%。固定资产投资、规模以上工业增加值等9项指标也都实现了翻番。我市先后被授予全国科技进步先进市、全国双拥模范城、全国农民健身工程建设先进市、群众体育先进市和全国白内障无障碍市。50个单位或单项工作进入全国先进行列。全省县域经济工作现场会在我市召开，县域经济发展成效被中央和省级媒体誉为“黄冈现象”。

　　过去五年，是重点项目建设突飞猛进的五年。大广高速、武英高速、武麻高速和鄂东长江大桥建成通车，武汉至黄冈城际铁路和黄冈长江大桥、黄鄂高速公路、大别山旅游公路、黄州火车站扩建改造、麻竹高速、九江长江二桥北岸接线等重点项目动工兴建。长江堤防全线加固，达到抗御1954年型特大洪水标准。引进了一批国内外知名企业。建设了一批民生工程和社会事业项目。特别是仅仅用16个月的时间，完成了目前世界上主跨最长的公铁两用长江大桥——黄冈长江大桥前期工作，仅仅用128天时间完成了2个主塔墩62根基桩施工任务，创造了国内重点工程前期工作的“黄冈速度”和国际同类桥梁建设的新纪录！

　　过去五年，是工业经济快速成长的五年。食品饮料、医药化工、纺织服装、建筑建材、机械电子等五大产业总产值首次分别突破100亿元大关。湖北祥云、兴业炉料、大别山电厂、华新（武穴）水泥、黄冈伊利、鸿路钢构、李时珍医药集团等7家企业年产值过10亿元。建成了全球最大的核黄素生产基地、全国最大的窑炉生产基地、中部地区钢构产业基地和华中地区最大的乳制品生产企业。医药产业和建筑业规模居全省第二位。拥有中国名牌2项、中国驰名商标2件、中国地理标志保护产品17个、湖北名牌36个、湖北省著名商标35件。这些都已成为“黄冈制造”的闪亮名片！

　　过去五年，是城乡面貌发生巨大变化的五年。农村住房建设、城中村改造和环境综合整治取得突破性进展，城镇建设和工业园区开发齐头并进，城乡基础设施全面完善，城镇建成区面积达到422.3平方公里，比2005年扩大100.2平方公里。黄冈市区主次干道基本完成了刷黑改造，园林绿化全面加强，尤其是遗爱湖公园的建设，彰显了古城黄州的文化内涵，提升了城市的文化品味，得到了市民的普遍欢迎。黄冈的大地变绿了，环境变好了，城乡更美了！

　　过去五年，是群众得到更多实惠的五年。城镇居民人均可支配收入和农民人均纯收入分别达到12836元和4634元，年均增长15.2%和11.9%。城乡居民人均住房面积分别达到44.4平方米和42.2平方米，比2005年增加15.2平方米和18.6平方米。家用小汽车五年新增4万辆。基本养老、医疗、失业、工伤、生育五项社会保险参保人数达到286.5万人次，城镇和农村低保人数分别达到14.1万和35.8万，新型农村合作医疗基本实现全覆盖。五年来，在推进老区建设的伟大实践中，全市人民的思想观念发生深刻变化，干事创业氛围更加浓厚，创先争优的激情空前高涨。黄冈，已经站在推进跨越式发展新的历史起点上！

　　五年来，我们主要做了以下工作：

　　（一）着力推进经济结构调整，经济平稳较快发展。认真贯彻国家宏观调控政策，从黄冈实际出发，把工作着力点放在做大总量、优化结构、转变发展方式上，注重速度与结构、质量、效益的统一，推动三次产业协同发展。工业经济快速成长，建成经济开发区和工业园区33个、乡镇工业集中区39个、省级重点产业集群7个、规模以上工业企业1577家、年产值过亿元企业156家。2010年，全市规模以上工业增加值达到245.7亿元，年均增长27.6%。高新技术产业增加值由2005年的11.4亿元增加到51.9亿元，年均增长35.5%。现代农业建设全面推进，各类农业产业化经营组织发展到2155个，农民专业合作社1274家，规模以上农产品加工企业492家。水产品年加工能力达到6.5万吨，出口创汇2500万美元。粮食连续七年增产，奶牛存栏2.4万头，多种经营基地面积690万亩。整耕机械化水平提高到58.3%。完成人工造林139万亩，退耕还林23万亩。全市农业增加值由2005年的115.5亿元增加到237亿元，年均增长15.5%。服务业加快发展，“万村千乡市场工程”、“新网工程”全面推进，建成农家店3437家，村级综合服务社1266家。金融、保险、通信、石油、烟草、房地产等行业长足发展，物流、商贸、餐饮酒店业提档升级，旅游业进入规模发展阶段，创建旅游强县1个、旅游名镇2个、旅游名村13个，新建4A级景区8个。2010年全市接待游客819万人次，实现旅游收入44亿元。

　　（二）着力加强基础设施建设，发展后劲进一步增强。深入开展“大干项目年”、“大干项目提升年”活动，切实加强城乡基础设施和基础产业建设。五年完成投资2111.6亿元，是“十五”时期的3.7倍。累计争取中央和省预算内投资51.1亿元。交通建设，完成投资244亿元，高速公路通车里程达到480公里，居全省第二位，率先实现了县县通高速；建设通村油路12702公里，实现了100%乡镇和行政村通油路（水泥路）、95%的行政村通客车。水利建设，争取国家和省投资31.8亿元，完成了205.7公里长江干堤、60.2公里长江回水堤防整险加固工程，除险加固百万立方米以上病险水库106座，更新改造大型泵站16座。取得了2010年抗洪救灾斗争的全面胜利。能源建设，建成了大别山电厂、白莲河抽水蓄能电站、鄂东500千伏环网和天然气工程。实施农村清洁能源入户工程22.3万户。坚持高起点规划，高水平建设，高效能管理，优化城市布局，提升城市功能，改善城市环境。市区累计投入城建资金25亿元，基本完成了“一湖二十八路”建设工程，启动了市博物馆等五大社会事业项目建设。推动城市管理创新，深入开展“四城联创”，集中治理违法建设、噪声污染等问题，更新出租车和公共汽车，成功创建省级卫生城市。县市城区总规编制完善率、详规覆盖率达到100%和70%，建制镇、乡集镇和村庄规划覆盖率分别达到83.3%、68.5%和46.5%。城镇建设完成投资75亿元，县市城区、小城镇和新农村建设全面加强。

　　（三）着力推动体制机制创新，改革开放取得明显进展。农村各项改革不断深化，集体林权制度改革验收达标，农村建房规划和用地管理得到加强，土地流转326万亩，林地流转206万亩，新建高标准农田60万亩。国有企业改制基本完成，国有资产监管体系逐步建立，财税体制改革进一步深化，集中财力解决了一些多年积累的矛盾和问题。投资、金融体制改革深入推进，金融机构各项贷款余额五年净增247.5亿元，市县两级全部进入全省最佳金融信用单位。英山农村合作银行正式挂牌。融资平台建设与开发性金融合作不断深化，经营性土地“招拍挂”全面推行。市县两级政府机构改革顺利完成，事业单位改革深入推进。价格和粮食流通体制改革取得新的进展。毫不动摇地支持民营经济发展，全市个体工商户发展到18.5万户，私营企业发展到1.1万家。外商投资企业增加到329个。积极推进武汉城市圈“两型”社会建设综合配套改革，加强生态建设和环境保护，创新推行河湖库长制，启动黄冈长江生态文明示范带建设，建成13座污水处理厂、11个垃圾处理工程和大别山电厂脱硫装置，依法关闭高耗能高污染企业110家，圆满完成了“十一五”节能减排任务。坚持以开放促改革、促发展。持续推进大招商活动，积极承接产业转移，累计引进招商引资项目3677个，其中亿元以上项目314个，总投资2068亿元；累计利用外资5亿美元，年均增长21.6%；累计实现外贸出口14.9亿美元，年均增长5.8%。成功举办了全国基础教育黄冈论坛、首届中国·黄冈大别山旅游节、第六届第七届湖北省黄梅戏艺术节和全市第二届运动会等重大节会活动。成功实现了国际友城关系建设的新突破。

　　（四）着力推动经济社会统筹发展，社会事业发展水平不断提升。围绕打造黄冈教育品牌，优化教育布局，深化教学改革，大力实施农村初中改造、校舍安全工程和农村合格学校建设，推动城乡教育均衡发展。全市小学、初中入学率保持在99%以上，高考本科上线率全省领先，职业教育年招生人数达到4万人，高等教育实现新发展。大力推进科技进步，深入开展市校市厅合作，实施科技计划项目395项。加强医疗卫生服务体系建设，建成乡镇卫生院68个、村卫生室121个，6个县市区实施了国家基本药物制度。农村新型合作医疗参合率达到97.8%。成功应对了甲型流感、手足口病等突发公共卫生事件。加强民生基础设施建设，黄冈市社会福利中心等一批民生项目投入使用。大力发展文化事业和文化产业，建成乡镇综合文化站124个，农家书屋3026个，建设了市文化中心、黄麻起义和鄂豫皖苏区纪念园等一批重点工程，创作了《李四光》、《东坡》等一批文化艺术精品。成功申报全国重点文物保护单位6处，国家级非物质文化遗产8项。影视基地建设和影视制作实现重大突破，电影《黎明行动》荣获国家“五个一工程”奖。统计、调查、气象、防震、民族、宗教、方志、档案、保密、经研、对台、侨务等工作得到加强，妇女、儿童、老龄事业健康发展。

　　（五）着力解决事关群众切身利益问题，人民群众生活水平不断提高。五年来，市政府承诺并完成了50个方面101件为民办实事项目。累计增加城镇就业人员25万人，就业培训52.7万人次，帮助就业困难人员和零就业家庭人员实现就业5万人，城镇登记失业率控制在4.4%以内。连续5年以年均10%的增幅提高了离退休人员的养老金待遇。团风县、黄梅县被列为全国新型农村社会养老保险试点。集中供养五保对象2.4万人。为636名0-6岁贫困残疾儿童实施抢救性康复救助，免费为3700名贫困白内障患者实施复明手术。建设廉租房等保障性住房29万平方米，住房公积金归集率、增值率不断提高。建成安全饮水工程2495处，223.8万农村人口喝上了自来水。大力推进扶贫开发，对1162个重点贫困村实施整村推进，累计投入扶持资金14亿多元，搬迁扶贫1万户，改造农村危房1.7万户，解决了40万低收入人口的脱贫问题。

　　（六）着力加强精神文明和民主法制建设，和谐黄冈建设迈出新步伐。深入开展群众性精神文明创建活动，公民思想道德素质和城乡文明程度明显提高。深入推进法治政府、服务政府、廉洁政府、效能政府建设，各级政府执行力和公务员能力建设得到加强。认真执行市人大及其常委会各项决议并向其报告工作，坚持向人民政协通报情况，自觉接受监督。密切联系工会、共青团、妇联和工商联等群众团体，广泛听取社会各界意见和建议。扩大基层民主，完善政务公开，保障人民依法行使民主权利。创新完善综治维稳信访联动机制，及时化解社会矛盾。加强社会治安综合治理，严厉打击各类违法犯罪。“法务前沿”工程在全国推广，社会治安综合治理视频建设等工作走在全省前列。加强应急管理，妥善处置了一系列突发事件。深入开展安全生产“三项行动”，安全生产工作和消防工作连年获得省政府表彰。加大食品、药品、农资市场整治力度，建成一批国家级和省级产品质量检验检测中心。健全落实计划生育责任制和“一票否决制”，全面完成了省下达的各项责任目标。强化审计、监察工作，深入推进源头治理，严肃查处违纪违法案件，廉政建设不断加强。电子政务建设取得明显进展。国防动员、民兵预备役和人民防空工作进一步加强。积极支持红安革命传统教育学院建设发展。四川灾区援建、对口援疆和南水北调移民试点安置工作得到国家和省充分肯定。

　　刚刚过去的2010年，我们始终坚持保增长、促转型、惠民生，巩固和扩大了应对国际金融危机冲击成果，较好地完成了市三届人大五次会议确定的主要目标。全市地区生产总值比上年增长14.1%，工业增加值增长23.6%，全社会固定资产增长34.8%，社会消费品零售总额增长22.2%，全口径财政收入增长32.7%，其中地方一般预算收入增长21.2%，单位生产总值综合能耗同比下降4.1%，化学需氧量和二氧化硫同比净削减2.78%和0.8%，城镇居民人均可支配收入增长13.2%，农民人均纯收入增长12.2%，居民消费价格指数103.5，城镇新增就业5.5万人。这些目标的全面完成，既实现了“十一五”规划的圆满收官，又为“十二五”发展打下了坚实的基础。

　　各位代表，五年取得的成绩来之不易，积累的经验弥足珍贵。这是省委、省政府和市委正确领导的结果，是全市上下团结奋斗的结果。在此，我谨代表市人民政府，向全市工人、农民、知识分子和各级干部，向人大代表、政协委员和各民主党派、工商联、无党派人士、社会团体，向驻黄部队和武警官兵、政法干警，向关心支持黄冈建设发展的海内外朋友们，表示崇高的敬意和衷心的感谢！

　　我们也清醒地认识到，当前经济社会发展还存在不少困难和问题：经济规模总量较小，市场主体偏少，综合实力不强；发展不平衡、不协调、不可持续的问题比较突出，农业基础仍然薄弱，工业竞争力和自主创新能力不强，服务业发展滞后，资源环境压力加大，影响发展的体制性机制性障碍尚未消除；教育、卫生、文化等社会事业和公共服务发展欠账较多，人民生活水平提高还不够快，改善城乡困难群众生产生活条件的任务很重；政府社会管理和公共服务职能还需进一步强化，执行不力、效率不高、自律不严的问题仍然存在。对于这些问题，我们一定高度重视，采取积极有效措施，努力加以解决。

　　二、“十二五”时期经济社会发展的总体要求

　　“十二五”时期，是黄冈发挥优势、加快发展的战略机遇期，是深化改革开放、转变发展方式的攻坚时期，是推进跨越式发展、全面建设小康社会的关键时期。根据党的十七届五中全会、省委九届九次全会和市委三届八次全会精神，市政府经过深入调查研究，认真听取各方意见，制定了《黄冈市经济和社会发展第十二个五年规划纲要（草案）》。

　　《纲要（草案）》对“十二五”时期我市经济社会发展环境和条件进行了全面深入分析。从外部环境看，中央促进中部地区崛起战略深入实施，武汉城市圈“两型社会”综改区建设扎实推进，大别山革命老区经济社会发展试验区建设即将启动，使黄冈处于多种比较优势的叠加期，发展的宏观环境十分有利。从内部条件看，通过全市上下长期不懈的努力，增强了体制机制活力，培育了一批工业园区和市场主体，开工建设了一批事关长远发展的重大项目，我市进入了城镇化加快推进的关键时期、产业结构优化升级的关键时期、基础设施完善提高的关键时期、城乡居民生活质量水平全面提升的关键时期。我们有条件、有责任、也有能力，在未来五年继续抢抓机遇、后发赶超，实现更好更快更大的发展。

　　“十二五”时期全市经济社会发展的指导思想是：高举中国特色社会主义伟大旗帜，以邓小平理论和“三个代表”重要思想为指导，深入贯彻落实科学发展观，以转变经济发展方式为主线，以深化改革开放为动力，以保障和改善民生为出发点，全面实施“一区两带”战略，大力推进新型工业化、新型城镇化、农业现代化，奋力推进跨越式发展，全力打造大别山革命老区经济社会发展试验区、武汉城市圈新兴产业基地、全省绿色农产品生产加工基地、全国红色文化旅游基地，为把黄冈建设成为现代化大城市奠定坚实基础。

　　未来五年全市经济社会发展的总体目标是：

　　——经济平稳较快发展。力争主要经济发展指标高于全国、全省平均水平，高于“十一五”发展水平。地区生产总值年均增长13%，力争经济总量和人均水平在“十一五”末基础上实现翻番。全社会固定资产投资年均增长25%、社会消费品零售总额年均增长18%、外贸出口年均增长15%以上、地方一般预算收入年均增长15%以上。

　　——经济结构进一步优化。到“十二五”末，全市二、三产业占GDP比重分别达到44%和36%左右。高新技术产业增加值占GDP的比重达到8%左右。城镇化率达到46%以上。

　　——城乡居民收入较快增长。低收入者收入明显增加，中等收入群体明显扩大，贫困人口显著减少，人民生活质量和幸福指数不断提高。建设城镇保障性住房5.95万套。城镇居民人均可支配收入和农民人均纯收入年均分别增长12%以上和10%以上。

　　——社会事业全面进步。城镇新增就业22万人，城镇登记失业率控制在5%以内。到“十二五”末，城镇参加基本养老保险达到56万人，城乡三项医疗保险参保率达到95%。人口总量控制在770万人以内。九年义务教育巩固率达到97%，高中阶段教育毛入学率达到95%以上。

　　——生态文明建设明显加强。森林覆盖率达到45%以上。耕地保有量569.7万亩。完成省下达的节能减排指标。

　　——改革开放不断深化。加快形成有利于科学发展的体制机制，经济社会发展的动力和活力进一步增强。加强政府自身建设，全面完成法治政府建设任务。

　　实现上述奋斗目标，重点把握好以下七个方面：

　　（一）把建设大别山革命老区经济社会发展试验区、推进跨越式发展作为“十二五”发展的主旋律。坚定不移地把跨越式发展写在黄冈“十二五”发展的旗帜上，把推进跨越式发展作为解决黄冈所有问题的关键、基础和总钥匙，始终保持清醒头脑，增强忧患意识，聚精会神搞建设，一心一意谋发展，努力在新一轮竞争中抢占制高点、增创新优势、实现新跨越。坚定不移地把建设大别山革命老区经济社会发展试验区，作为实现跨越式发展的历史使命、政治责任和主要平台，按照建设“红色大别山、发展大别山、绿色大别山、富裕大别山”的总体要求，以统筹城乡经济社会发展为目标，以促进居民收入增长为出发点和落脚点，以解放思想、改革创新为动力，突出重点、分步推进，力争经过3至5年的努力，把大别山革命老区经济社会发展试验区基本建成推进黄冈跨越式发展的重要引擎和全国革命老区经济社会发展示范区。

　　（二）把实施“一区两带”战略作为“十二五”发展的主要载体。围绕高昂市区龙头、率先推进黄冈武汉同城化，加快构建临港产业、综合交通、综合物流三大体系，实现基础设施、产业布局、城乡建设、区域市场、生态环保五个一体化，努力把黄冈临港经济区建设成为市域经济发展的龙头、武汉城市圈“两型”社会建设的先行区。围绕培育大工业、发展大农业、搞活大物流，加快沿江现代产业密集带、新型城镇连绵带、长江生态文明示范带建设步伐，努力把黄冈长江经济带发展成为特色鲜明、竞争力强、国内一流的沿江产业聚集带、承接产业转移的主要基地和长江中游北岸明星城市群。围绕“大别山水、人文黄冈”，全力打造大别山旅游品牌，突破性发展现代旅游业，努力把黄冈大别山红色生态文化旅游经济带建设成为展示黄冈魅力的景观带、加快脱贫致富的示范带和国内外知名的旅游目的地。

　　（三）把加快转变经济发展方式作为“十二五”发展的主线。牢固树立“产业第一”的理念，坚持战略性调整与适应性调整并重，存量调整与增量优化并重，产业结构优化与产业层次升级并重，进一步发展先进制造业，大力培育发展高新技术产业和战略性新兴产业，全面推进农业现代化，加快现代服务业发展，加强基础设施和基础产业建设，加快构建三次产业协同带动的现代产业体系。积极扩大消费需求，保持投资持续增长力度，加快推动出口增长，努力实现经济增长协调拉动。大力推进新型城镇化，突出黄冈城东新区建设，拓展发展空间，提升城市形象；加强县市城区和小城镇建设，全面推进新农村建设，促进城乡一体化发展。扎实推进“两型”社会建设，强化节能减排，走绿色发展、和谐发展之路。

　　（四）把深化改革开放作为“十二五”发展的主要动力。以更大决心和勇气，大力推进重点领域改革。扎实做好农村土地管理制度改革、农村综合改革、农产品流通体制改革、行政管理体制改革、国有企业改革和国有资产监管体制改革、投融资体制改革、财税体制改革以及科技、教育、文化、卫生、体育等社会事业单位分类改革工作。实施更加积极主动的开放战略，将“走出去”与“引进来”有机结合起来，着力提高招商引资质效和水平，不断拓展对外开放的深度和广度。

　　（五）把统筹经济社会发展作为“十二五”发展的基本要求。加快科技创新体系建设，走创新驱动之路，最大限度地发挥自主创新对全市经济社会发展的支撑作用。认真贯彻落实教育改革发展中长期规划，优化教育发展环境，不断加大教育投入，打造黄冈教育品牌，建设教育强市。以保基本、强基层、建机制为重心，积极推进医药卫生体制改革，建立四位一体、覆盖城乡的基本医疗卫生制度。进一步做好人口和计划生育工作，切实保障妇女儿童和残疾人的合法权益。以深化文化体制改革为动力，推动文化大发展大繁荣，建设文化强市。统筹推进各类人才队伍建设，推进人力资源大市向人才强市转变。坚持更高标准，积极推动各项社会事业发展。

　　（六）把保障和改善民生作为“十二五”发展的根本目标。着力增加民生净福利，使人民群众生活得更加幸福、更有尊严。在增加城乡居民收入上，实现“两个同步”：居民收入增长与经济发展同步，劳动报酬增长和劳动生产率提高同步。在完善社会保障体系方面，实现“三个提高”：提高保障水平、提高覆盖面、提高社会保障机制的科学化水平。在加大投入方面，实现“四个增加”：用于改善民生的投入总量、增量、增幅、占比逐年增加。

　　（七）把推进和谐黄冈建设作为“十二五”发展的重要保障。继承黄冈老区传统，加强精神文明建设，构建社会主义核心价值体系，不断提高城市文明程度。加强和创新社会管理，完善利益协调、诉求表达、矛盾调处和权益保障机制。健全突发事件应急管理体制，提高防灾减灾能力。加强法制宣传教育，深入开展法治黄冈、平安黄冈建设，增强公共安全保障能力。

　　三、扎实做好2011年工作，努力实现“十二五”良好开局

　　2011年是实施“十二五”规划的第一年，做好今年各项工作具有十分重要的意义。今年全市经济社会发展的主要预期目标是：地区生产总值增长13％左右；规模以上工业增加值增长20%以上；全社会固定资产投资增长25％以上；居民消费价格指数涨幅控制在4％左右；社会消费品零售总额增长18％；地方一般预算收入增长15％以上；外贸出口增长15%以上，实际利用外资增长10%；单位生产总值综合能耗下降和主要污染物减排完成省下达指标；城镇居民人均可支配收入增长12％，农民人均纯收入增长10％；城镇登记失业率控制在4.6%以内；人口自然增长率控制在6‰以内。

　　第一，全力推进大别山革命老区经济社会发展试验区建设，高标准做好开局之年的各项工作。抓紧编制试验区发展规划。按照统筹区域协调发展的目标要求，衔接全省、全市“十二五”规划，对接武汉城市圈和环大别山发展总体规划，面向全国、适度超前，高水平编制黄冈大别山城乡空间格局、产业布局、基础设施、社会事业、公共服务、社会管理、市场体系、生态环境建设等试验规划，以科学规划引领试验区发展。抓紧策划和争取试验项目。立足黄冈实际，着眼长远发展，围绕完善试验区高速公路网、快速干线交通网、农村公路网、低碳水运网、综合交通运输网，加强水利基础设施建设，增强大别山地区抗御自然灾害能力，加快提升城乡道路、供电、供水、排污设施能力和人居环境，精心策划和争取实施一批大项目、好项目。抓紧争取创新试验政策。在建立多元化投入机制、土地利用机制、环境保护和生态补偿机制、扶贫开发机制、人才培养机制等方面，争取全域推进、先行先试，争取国家和省更多优惠政策和资金支持。抓紧承办湖北大别山扶贫协作优势产业推介活动，精心组织招商对接洽谈，争取有一批大型企业集团到黄冈投资落户。

　　第二，扩大投资规模，加强重点项目建设。进一步加大投资力度，力争投资总额突破1000亿元。着力推进重点项目建设。在高质量完成在建项目续建和收尾工作的同时，继续做好项目储备和推进工作，突出抓好283个续建和新开工亿元重点项目，特别是列入全省重点建设计划的林浆纸一体化、城际铁路和黄冈长江大桥、黄鄂高速、武汉新港江北铁路黄冈段等21个重大项目建设，全面完成大别山旅游公路建设任务，扎实推进浠水核电、抽水蓄能电站、风力发电、黄冈电厂、大别山电厂二期工程、随麻安铁路、黄冈机场等重大项目前期工作。进一步提升招商引资层次。组织实施好大型境内外重点招商活动，加强与世界500强、上市公司、大型央企民企的合作，支持企业引进战略投资者。改进产业招商办法，建立选商择资综合评价体系，有针对性地引进产业链各环节的重点企业和配套企业，引导资金投向先进制造业、现代服务业和生态高效农业领域。完善重点项目落地跟踪服务机制，优化投资发展环境。

　　第三，突出工业主导地位，增强经济发展竞争力。继续加快百亿园区、百亿产业、百亿企业“三个一百”工程建设步伐，促进食品饮料、医药化工、纺织服装、建筑建材、机械电子等传统产业向更高层次迈进。继续实施“中小企业成长工程”和“市场主体增量行动”，不断提高工业投资的比重，力争新增加100家规模以上工业企业，新上50个投资过亿元的工业项目。加强开发区和工业园区建设，着力壮大产业集群，打造具有黄冈特色的板块经济。继续推进质量兴市，实施精品名牌战略，深化市厅市校合作，培育一批名牌产品和拥有知名品牌的大型企业。重点发展生物医药、新材料、节能环保等高新技术产业，发展壮大造船、窑炉、电子、钢构等新兴产业。加强和改善对企业的服务，继续实行重点企业专班服务和领导挂点服务，扎实开展大企业直通车服务。建立和完善中小企业服务体系，搭建创业辅导、融资担保、信息网络、人才培训等服务平台。加强经济运行监测，抓好生产要素的组织协调，力保煤电油气运有效供给。

　　第四，加快发展现代农业，努力建设社会主义新农村。加强农田水利建设。抢抓中央加快水利改革发展的重大政策机遇，着力抓好防洪薄弱环节的整治，加大中小河流治理和中小水库除险加固、山洪地质灾害防治、易灾地区生态环境综合治理力度。抓好高标准农田建设和小型农田水利建设，重点推进大中型灌区续建配套、节水改造和“小农水重点县”建设。加强农业气象服务和灾害防御体系建设。提高农产品加工水平。通过政策引导、市场推动、招商引资等办法，做大做强龙头企业，不断提高农产品转化率、增值率、农产品加工产值占农业总产值的比率，建设一批国家和省级农产品加工示范园区，形成一批技术含量高、具有地域特色的名牌农产品。提高农产品供给能力。全面落实国家支农惠农政策，加大支农资金整合力度，集中连片建设一批现代农业产业带，积极创建农业标准化示范区，形成一批专用、优质、稳定的农产品加工原料生产基地。大力推进农业机械化，不断提高农机保有量和装备水平。加强重大动物疫病防控，提高畜产品质量安全水平。落实“菜篮子”市长责任制，抓好新一轮“菜篮子”工程建设。进一步改善农村生产生活条件。整体推进农村土地整治工作，加强农村电网改造、安全饮水、乡村公路、清洁能源等设施建设，完善基层医疗卫生、文化、体育和商品购销服务。深入推进开发式扶贫，以整村推进和连片开发为平台，实现农村低保制度与扶贫开发政策有效衔接，再解决8万贫困人口脱贫问题。

　　第五，加快发展现代服务业，增创经济发展新优势。加快发展旅游业。以培育发展旅行社为突破口，充分发挥其聚集旅游要素作用，制定扶持激励政策，培育和引进骨干旅行社，拓展地接业务，强化旅游设施建设、产品开发、市场营销和旅游人才队伍建设，全面提升黄冈旅游品牌影响力和旅游业整体发展水平。加快发展金融业。加强金融生态环境建设，努力扩大信贷投入；加大农村信用社改革力度，力争全面完成农村合作银行组建任务；优化金融市场主体结构，争取再引进1至2家股份制商业银行；支持发展商业保险，重点扩大“三农”保险覆盖面；积极做好小额贷款公司申报、组建和各类融资平台规范发展与债券发行工作，着力推进企业上市，争取1至2家企业完成上市申报工作。加快发展商贸流通业。加强市级和县级商业中心建设，支持城市商业网点改扩建项目，做好“农超对接”工作，提升物流业发展水平。继续实施家电下乡和以旧换新政策，推动农村商业连锁经营和统一配送，积极发展电子商务、网络购物等新型消费业态。加强粮油等重要商品物资购销和储备工作。积极争取全省服务业综合配套改革试点。继续为通信、石油、烟草、房地产业加快发展创造良好条件。加快发展文化产业。实施一批文化产业项目，扶持一批重点文化企业，打造一批文化产业基地。编制黄冈名人文化资源开发、保护、利用专项规划，启动一批名人文化开发保护工程。深入整顿和规范市场秩序。加大对乱收费乱涨价的整治力度，发挥价格基金调控作用，保持市场价格基本稳定。严厉打击侵犯知识产权和制售假冒伪劣商品行为，切实维护消费者权益。

　　第六，推进市区一体开发，加快县域经济发展。按照统一规划、统一管理、统一投资、统一建设、统一招商、统一征税的原则，进一步理清市区发展思路，创新体制机制，整合资源，凝聚力量，加快形成市区一体、资源共享、产业聚集、和谐发展的新格局。按照“政府主导、市场运作”的原则，规范壮大市城投公司，建立城东新区开发和市区一体开发投融资平台，拓展间接融资，引进战略投资，争取政策投资，激活民间投资，为市区建设发展注入新的活力。抓紧编制完善城东新区规划等工作，加快五大社会事业工程建设步伐，继续推进遗爱湖综合治理保护工程和“数字黄冈”建设，新建一批市政工程。持续开展“四城联创”，提升城市执法管理水平，努力创建省级环保模范城市和省级园林城市。进一步完善激励竞争机制，加快发展县域经济。以工业经济为主导，以园区建设为突破口，加大基础设施建设和招商引资工作力度，集中力量培育紧密度高、产业延伸链条长、市场竞争力强的产业体系，打造一批财税贡献大、发展前景好的骨干企业。进一步完善城镇规划体系，突出抓好县城和中心镇建设，大力发展小城镇，构筑县城、重点镇、小城镇梯次推进的城镇化发展格局。深入开展村镇建设“八个一”竞赛活动，抓好“楚天杯”、“大别山杯”创建活动。继续推进20个生态文明镇、200个生态文明村和特色村镇建设。认真落实国务院关于鼓励引导民间投资“新36条”，进一步改善投资创业环境，积极推动全民创业，鼓励引导返乡创业，支持民间资本进入基础产业、基础设施、市政公用事业、社会事业、金融服务等领域。

　　第七，深化改革试验，推动体制机制创新。继续推进农村各项改革，稳定和完善农村土地承包经营制度，稳步推进土地适度规模经营和土地承包经营权流转，建立健全农村集体“三资”监管代理工作机制，进一步扩大城中村、城郊村、园中村产权制度改革创新试点，完善农村公益性服务“以钱养事”机制，扩大村级“一事一议”财政奖补试点范围。巩固完善集体林权制度改革成果。加快农垦改革发展，发挥国有农场在现代农业建设中的示范带动作用。大力发展农村专业合作组织，建立完善农业服务体系。落实国家深化资源要素价格改革部署，促进资源产权和产品自由流动。深化财税体制改革，调整优化财政支出结构，加强部门预算管理，新增财力优先保障“三农”、社会事业和民生工程投入；坚持依法治税，强化财税收入征管，确保完成财政收入任务，实现财政收支平衡。按照“股权多元化、经营企业化、运作市场化”的要求，做大做实各级各类融资平台。以履行出资人职责和实现国有资产保值增值为重点，推动国有资本向关键领域和民生行业聚集。深化教育、文化、医疗卫生体制改革，完成年度重点改革任务。大力实施开放先导战略，加快转变外贸发展方式，加强出口基地建设，增加自有品牌、自主技术出口比重，突出抓好重点产业、重点企业、重点产品的出口工作。加强对外经济技术合作，加大外派劳务基地建设力度，努力提高引资引智工作水平。

　　第八，大力推进节能减排，强化生态环境保护。进一步完善节能减排管理机制和体系，严格执行节能减排目标责任制和行政首长负责制，建立健全以减排为重点的“倒逼传导”机制，确保完成省政府下达的节能减排任务。抓好“双超”、“双有”企业强制清洁生产审核工作，全面实行排污许可证制度。对重点耗能企业实行分级管理，大力发展节能产业，鼓励节能投资机构以合同能源管理方式为企业进行节能改造。加快实施城镇污水垃圾处理项目，加强环保设施的正常运营管理，坚决杜绝建而不用现象，不断提高城镇生活污水、垃圾处理率。全面推进循环经济发展，加快建设城镇矿产示范基地、再制造产业、餐厨废弃物资源化工业、农业循环经济示范、产业园区循环化改造等五项工程，切实加强对石材行业的规范整治，促进环境保护和资源循环利用。继续实行河湖库长制，实施水生态修复工程，保障城乡居民饮水、用水安全。全面加强农村环境整治工作，重点抓好连片整治示范区建设，确保第一批37个试点村通过国家和省验收。

　　第九，切实保障和改善民生，加快发展社会事业。稳定和扩大就业，以高校毕业生、农村转移劳动力、城镇就业困难人员和退役军人为重点，实施更加积极的就业政策，发展就业容量大的服务业、劳动密集型产业，多渠道开发就业岗位，促进充分就业。进一步完善劳动合同、集体合同制度和劳动关系三方协调机制，加大劳动保障执法监察力度，维护劳动者合法权益。建立工资集体协商制度，健全工资支付保障制度，建立稳定的职工收入增长机制。坚持广覆盖、保基本、多层次、可持续的方针，进一步健全社会保障制度体系，切实组织好新农保试点，全面推进城镇企业职工养老保险省级统筹，医疗、失业、工伤和生育保险地市级统筹。健全城乡居民最低生活保障制度，做到应保尽保。进一步提高保障水平，落实企业退休人员基本养老金调标、医疗保险提待遇等工作。加强保障性安居工程建设和房地产市场调控，加强住房公积金归集、使用和监管，引导住房合理消费。大力发展教育、卫生、文化等社会事业。编制和落实中小学布局调整和发展规划，加强学校标准化和师资队伍建设。改善农村和贫困地区办学条件，加快实施小学、初中薄弱学校改造和中小学校舍安全工程。重视发展学前教育。加强中等职业教育基础能力建设，推进中职院校资源整合，建设职教园区。支持提升高等教育办学水平。加强基层医疗卫生服务体系建设，再改造建设一批县级医院和乡镇、社区卫生服务设施，力争消灭乡镇卫生院D级危房。抓好艾滋病、结核病、血吸虫病等重大传染病的防治工作。加强县级电影院、农家书屋、乡镇综合文化站、文化信息共享工程等公共文化服务体系建设，深入开展送戏、送书、送科技下乡活动。不断提高广播影视数字化、网络化水平，促进“三网融合”。严格执行人口和计划生育政策，建立完善利益导向机制，健全计生服务体系，依法打击“两非”行为，提升人口计生工作水平。办好第三届全市运动会。继续实施“农民体育健身工程”。创新社区管理和服务体制，扩大农村社区建设试点范围，推进社区建设全覆盖。依法做好第八届村委会换届选举工作。严格统计和调查监测评价考核。继续做好二轮修志和年鉴编撰工作。培育壮大老龄服务事业和产业。健全残疾人社会保障和服务体系。依法管理民族宗教事务，加强档案信息资源开发，认真做好外事、侨务、对台、保密等工作。

　　第十，加强政府自身建设，努力提高科学发展的能力和水平。自觉接受人民代表大会及其常委会的法律监督，接受人民政协的民主监督，认真听取各民主党派、工商联、无党派人士和人民团体的意见，不断改进政府工作。全面推进依法行政，积极推进基层依法治理，完善行政执法程序，规范行政执法行为，更加注重保护人民合法权益。全面推进政务公开，重点推进财政预算、公共资源配置、重大建设项目、社会公益事业等领域的信息公开。进一步规范市县乡村四级行政服务网络，深化行政审批制度改革和投资体制改革，加快电子政务和电子监察建设，全面推进“阳光审批”和网上审批，严格实行招投标“五统一”。围绕建设法治黄冈、平安黄冈，进一步加大社会治安综合治理和安全生产工作力度，创新推进应急管理工作，依法打击各类违法犯罪活动，有效应对突发公共事件，夯实消防安全“防火墙”，提高危机管理和抗风险能力，促进社会和谐稳定。强化产品质量、食品药品安全和特种设备安全监管。加强国防动员、民兵预备役和人民防空工作，重视和支持驻黄部队建设，扎实做好全国双拥模范城（县）创建工作。健全政府“确责、履责、问责”责任体系，狠抓各级公务员执行能力建设，注重深入基层、深入群众、深入一线，有效推进政策落实、项目落地、民生落户。加强政府工作人员的学习培训，着力提高各级公务员推动科学发展和加快转变经济发展方式的能力。加强廉政建设和反腐败斗争，认真落实党风廉政建设责任制，严格执行领导干部“一岗双责”，加强行政监察、政务督查和经济责任审计，加大案件查处力度，让权力在阳光下运行。

　　各位代表！今年市政府继续从群众最关心最直接最现实的利益问题入手，办好十件惠民利民实事：（1）全市城镇新增就业4万人，培训劳动力30万人次。（2）建设保障性住房工程40万平方米。（3）再解决农村10万人饮水安全问题；实施清洁能源入户工程6万户；改造农村危房8100户（其中改造农村贫困残疾人危房600户）；实施农村环境连片整治示范工程，每个县市区选择5至10个村作为重点片区，建设1至2个重点村。（4）实施六大食品安全工程，2年内每个县市区建成1个农产品检测中心、1个豆制品集中加工小区、1个生猪定点机械化屠宰场、1条餐饮单位笑脸服务标识管理示范街、1个学校粮油集中配送站、1个餐厨垃圾规范管理工程。（5）全市新增出租车200台、客车160台，更新出租车120台、公交车120台、客车300台；发展省际和市际客运线路8条、县际客运线路5条、农村客运线路20条。（6）在所有乡镇卫生院、社区卫生服务中心实施基本药物制度，对484种基本药物实行零差率销售。（7）建成义务教育阶段标准化学校350所，新建乡镇公办幼儿园50所。（8）为260名0-6岁贫困残疾儿童实施抢救性康复手术。（9）继续为困难群众和农民工提供法律援助。（10）市区建成无害化垃圾处理场一期工程，新建垃圾中转站5座。

　　各位代表！黄冈未来五年的发展蓝图已经绘就，我们满怀信心迈上新的征程。让我们更加紧密地团结在以胡锦涛同志为总书记的党中央周围，在市委的领导下，深入贯彻落实科学发展观，紧紧依靠全市人民，扎实工作，奋勇争先，为开创黄冈改革开放和现代化建设的新局面而努力奋斗！
